# The Moral Justifications of Disability Discrimination in Health Care Allocation: An Experimental Assessment

**DOI:** 10.1007/s10728-025-00535-0

**Published:** 2025-08-22

**Authors:** Andreas Albertsen, Bjørn Gunnar Hallsson, Lasse Nielsen

**Affiliations:** 1 Aarhus University, Aarhus, Denmark; 2 University of Southern Denmark, Odense, Denmark

**Keywords:** Priority setting, Disability, Discrimination, Bioethics, Healthcare

## Abstract

Cost-effectiveness analysis (CEA) is a mainstay of contemporary health care priority setting. However, priority setting in reference to cost-effectiveness may discriminate against people with disabilities. The ethical literature on priority setting suggests that the permissibility of such discrimination varies with the reason why people with disabilities receive lower priority. In a vignette-based survey experiment (N = 1100) in the UK, we tested whether five justifications for prioritizing people without disabilities affect the views of the broader public on priority setting based on CEA. In our vignettes, a hospital denies a person with a disability treatment for a disease based on CEA, and respondents were asked to assess the moral permissibility of this. The vignettes varied in terms of the reason the hospital emphasized in the decision. We tested vignettes emphasizing lower expected lifespan, lower quality of life, higher costs of treatment due to disability, less efficient treatment due to disability, and lower productivity due to infrequent labor-market participation. Our study is an initial exploratory survey experiment, exploring participant’s responses to CEA with respect to disability. Discrimination against the patient with a disability was deemed impermissible across all experimental conditions, and there were no significant differences between the various reasons. This suggests a discrepancy between folk intuitions and those of many ethicists.

**Supplementary Information:**

The online version contains supplementary material available at https://doi.org/10.1007/s10728-025-00535-0.

## Introduction

Limited budgets and the emergence of expensive new medicines and treatments make priority setting an ever-present feature for contemporary societies [1, 2]. Across the world, there is a significant heterogeneity in how this is conducted [3], ranging from informal bedside rationing to the creation of priority-setting institutes or committees. A famous example of this kind of priority setting is the public consultancy conducted by the Oregon Commission in 1989 [4–6]. As one of the first institutes providing official guidance on health care priority setting and allocation of health resources, the National Institute for Health and Clinical Excellence (NICE) was founded in 1999 in the UK. Since then, different instantiations of similar institutions have spread across the globe. Prominent examples include New Zealand [7], Norway, Sweden [8, 9], Israel [10], the Netherlands [11], Canada [12], the United States, and Spain [13, 14]. While these differ in how they operate and make decisions, they all reflect a commitment to employ knowledge about the effect of medicine and treatment to decide the degree to which such a treatment should be used. In doing so, various factors, often including costs, are considered.

A critical aspect of such priority-setting measures has been introducing cost-effectiveness analysis (CEA), where the ratio between cost and benefits is calculated. While frequently employed, the role afforded to CEA in priority setting remains a source of controversy and discussion. A particular pertinent source of controversy, is the ability of such priority setting to avoid discrimination [15].

A prominent aspect here is the so-called disability discrimination problem [16–20]. The problem occurs because cost-effectiveness, on the one hand, is an integral part of a justifiable scheme for the allocation of health care resources. Still, on the other hand, it might lead to discrimination, which conflicts with strong anti-discrimination norms [21], and has been shown to have practical relevance [22]. In particular, it seems to discriminate against people with disabilities. The reason for the problem is that interventions and treatments, other things equal, are likely to generate more health effects when given to non-disabled than to disabled patients [23–25]. These effects are typically accounted for by standard measures such as Quality-Adjusted Life Years (QALY) or Disability-Adjusted Life Years (DALY), in which duration of time (e.g., 20 years) in a given health state (e.g. Arthritis) is multiplied by a quality- or disability-coefficient meant to capture the relevant reduction in quality of life, the health state involves. Therefore, cost-effectiveness and non-discrimination stand as potentially conflicting values in the debate on priority setting. Interestingly, this was, in fact, foreshadowed in the reactions to the Oregon priority setting exercise [1, 6, 26].

More recently, disability right advocates have highlighted a number of US-based cases where schemes for allocating critical care in face of the Covid-19 pandemic unjustly discriminated against people with disabilities in reference to cost-effectiveness criteria. The examples include policies that base priority decisions on judgement of quality-of-life or exclude patients with specific disabilities not related to the treatment from the pool of eligible recipients. Examples are, among others, from Alabama where guidelines called for hospitals to withhold ventilators from patients with “severe or profound mental retardation,” “moderate to severe dementia,” or “severe traumatic brain injury”, and from Utah’s recommendation to exclude patients with advanced neuromuscular diseases [22, 27]. These examples bear witness that the disability discrimination problem is not merely a philosophical construct or historical phenomenon but also a real, practical issue, and they demonstrate the need to explore the ethics of disability discrimination in more detail.

The above naturally invites the question of when and why (if ever) such discrimination against people with disabilities is justified. This is a significant question entertaining both philosophers and empirical scientists. In the philosophical literature, ethicists have discussed the disability discrimination problem intensively for the last decades. Still, more work needs to be done to investigate public opinion on the moral justifications for the discrimination. This is a crucial omission.

In this paper, we contribute to filling this gap with novel insights from an initial exploratory experimental study of the public’s reactions to different moral reasons for disability discrimination based on CEA. The experimental design allows us to study whether people’s judgments on the permissibility of disability discrimination in health care prioritization vary according to the moral reasons laid out as justifications. In order to make our results speak as directly as possible to the debates in the ethical literature, we have deliberately chosen a design which closely follows the kind of cases this literature often employs, although this choice comes at the cost of lower ecological validity. The results of our study indicate a broad rejection of cost-effectiveness-based priority setting when it disadvantages people with disabilities. This rejection is unaffected by which reason is emphasized as being the reason for the decision.

Our study aimed at informing the ethical debate on health care priority setting by investigating which reasons affect the moral judgments of laypeople. On a general level, it might be asked why ethical theory should take note of the broader public’s responsiveness to the reasons deemed weighty by ethical theorists when it comes to questions of the permissibility of healthcare discrimination. Indeed, some would deny that it should, appealing, for example, to the so-called expertise argument [28, 29] and submit that as laypeople are less trained and have less insight into the complex arguments and distinctions, the importance of what laypeople think should not be overstated. On the other hand, disagreement between ethicists and laypeople might indicate a failure of armchair theorizing to capture the complexities of moral judgment health-care settings. While it is clear that laypeople’s views do not decide normative questions on their own, we would maintain that they provide (defeasible) evidence that normative theorists should take into account [30]. Specifically, the consistency with which our participants denied the permissibility of disability discrimination due to cost-effectiveness regardless of justification seems to put at least some explanatory burden on defenders of this implication of CEA and each justification as to why the views of the broader public should not be considered.

The evidential weight of laypeople’s judgments might vary between topics [31]. There are several reasons to believe the disability discrimination problem of CEA is an area where folk intuitions are informative. One reason is that ethicists and philosophers often appeal to such intuitions in the literature when they present hypothetical cases and suggest that many would think in a particular way about the case. Second, because the disability discrimination problem is a case of genuine value conflict, in which there are sound reasons on both sides (i.e., cost-effectiveness and non-discrimination), philosophers should take account of how the public receives these reasons. Third, when the issue is of a political sort, as in this case, of prioritizations within the health care system, we need to be carefully aware that the reasons behind our priorities can be justified towards the public. It would be unwise to ignore the public’s assessments on these matters, if their continued trust in and support for healthcare priority-setting is essential.

After a brief presentation of the literature on disability and cost-effectiveness, the paper presents our methods, before laying out the results and discussing the implications and limitations of our study.

## The Disability Discrimination Problem

Roughly put, the disability discrimination problem arises because a cost-effective scheme for health care priority setting discriminates between patients’ claims for treatment on the ground that people with disabilities are less cost-effective to treat than people without disabilities. This poses a problem because many would find that such a priority decision is a case of unjust discrimination. Differential treatment based on membership of a group of disabled patients in reference to an allocation of goods seems to display wrongful (i.e., harmful or disrespectful) discrimination [32–34].^1^ Slightly crudely put, the attractiveness of cost-effectiveness in priority setting tracks a maximizing consequentialist (e.g., utilitarian) logic, whereas the unfairness of disability discrimination to a larger extent represents deontological morality. However, whether the discrimination is justified depends on the reasons offered as justification [36]. To inform whether such discrimination can be justified, we must consult the ethical literature on cost-effectiveness and disability discrimination.

Note that the disability discrimination problem, as we present it, is a philosophical rather than practical problem. The issue is not whether cost-effectiveness *analysis* is used by health economists and decision makers in a way that actually discriminates against people with disabilities. Evidence suggests that this is to a large degree not the case. Among other things, this is because cost-effectiveness analysis typically compares treatments in reference to added effect rather than absolute effect, and because standard measures of health-related quality of life uses large-data averages, which do not differentiate between people’s background condition [37]. The issue is, rather, when and why such discrimination would be morally justified by reference to cost-effectiveness (if it would ever be justified). This is by itself a relevant philosophical question and would still be so even if cost-effectiveness analysis is never used discriminatorily. We need to study the ethical relation between cost-effectiveness and discrimination to understand our reasons for finding disability discrimination problematic. In particular, if disability discrimination in reference to cost-effective health care priority setting is justifiable under some circumstances, according to the ethical literature, but cost-effectiveness analysis is very rarely used to discriminate in practice, it seems relevant to study the public’s reactions to those circumstances.

Most ethicists find the appeal to cost-effectiveness morally permissible, or even required, in priority setting scenarios with resource scarcity [1, 16, 38–40]. This does not imply that we ought always to prioritize to maximize cost-effectiveness, but merely that it is one important consideration that should be given a place in our priority setting deliberation. The inclination to give special weight to cost-effectiveness has been met with serious moral objections, in particular emphasizing the disability discrimination problem [18, 26, 41–43], but few take the criticism to imply that we should completely abandon cost-effectiveness.

The ethical literature clearly suggests that whether and the degree to which cost-effectiveness is permissible despite being discriminatory depends on the substantial justification. As ethicists have highlighted various justifications, we utilize this discussion to inform the presentation of various scenarios where discrimination might be deemed permissible. Importantly, our aim is not to take a particular stand on these different theoretical justifications (and in fact the authors have somewhat conflicting intuitions). Rather, the aim is to put these justifications to test by exploring convergence with public opinion.

First, one important factor highlighted in the ethical discussion is the assumption that disability reduces quality of life (e.g. in reference to QALY or DALY measures). This justification has been met with much criticism from philosophers and ethicists. One central objection comes from the so-called mere-difference view on disability. In brief, the mere-difference view holds that lives with disabilities are not intrinsically worse but merely different than non-disabled lives and that the disadvantage for disabled people primarily stems from the ableist construction of the societal environment [44]. Another related objection is the “linkage” or “double-jeopardy” problem which has been discussed (though not endorsed) by Kamm and Brock [18, 42]. The objection holds that it is unfair if the disadvantage of living with a disability should count twice by being transferred from everyday inconvenience into also receiving lower priority in health care priority setting.

Second, another relevant factor is that disability often also reduces life expectancy. Even if it could be unfair to discriminate based on reduced quality of life, it would be strange if cost-effectiveness should not be sensitive to how many lives a treatment would produce. Some theorists explicitly note that quantity of life intuitively seems a more promising justification for disability discrimination than quality of life [42, 45]. However, still, some are critical about life expectancy as a ground for disability discrimination. One central reason is that in any case, it seems improper to count in the disability as relevant for the deliberation of allocation of treatment when the disability has no relation to the condition the treatment is targeted against [18]. Another reason refers to fairness in the more general sense that the disadvantage of the disability should not count against the disabled because it is beyond their exercise of responsibility [46]. Still, the discussion suggests that some might be more sympathetic to reasons related to life expectancy than life quality as justifications for disability discrimination.

Third, there is a common thrust in the criticisms of priority setting based on the idea that the lives of people with disabilities are shorter or of lesser quality. In deciding whether to treat a person for a specific illness, it seems peculiar to take factors into account that are unrelated to and do not interact with our ability to effectively remove this illness but which, instead, arise because of the disability. However, understanding the criticisms in this way, of course, paves the way for a family of reasons why giving lower priority to people with disabilities might (sometimes) be justified. These pertain to situations where the presence of the disability either increases the costs of treatment or reduces its efficiency. It is a common theme in bioethical discussions that such a connection may make it more permissible to give lower priority to people with disabilities.

A fourth contender for justifying disability discrimination is the lower expected economic return from investment in disabled than non-disabled people. It is intuitive that allocating treatment to patients who are otherwise fully functioning will be a safer economic investment than if the treatment is given to a group of patients with reduced employability and work capability. However, health care is generally perceived to be a central social good independent of the return of investment.

Fifth, we can imagine an effect from comorbidity on perceived permissibility of disability discrimination. Because treatment of people with existing disabilities may in many cases be more complicated and more medically costly, we might expect a CEA-based justification for giving lower priority to people with disabilities. This is a different justification, as it refers specifically to medical costs and not the aggregate economic return more generally.

Finally, several studies find some public skepticism towards aspects of cost-effectiveness in priority setting [2, 25, 47, 48]. But so far, the literature has not tested whether this skepticism can be decreased by providing reasons it might be permissible to discriminate against the disabled based on CEA. Whether to expect convergence there is a difficult question. Perhaps medical ethicists working on cost-effectiveness and priority setting might be inclined to give special weight to the issues and factors of interest to their work. Moreover, the philosophical literature at times encounters and displays nuances of discrimination that may be of moral importance but that seem less relevant from the perspective of the general public. This suggests that we should expect public opinion to diverge from justifications prevalent in the ethical literature.

## The Present Study

We aimed at investigating public judgments of the moral permissibility of disability discrimination in health care provision based on CEA, and whether these judgments are affected by drawing attention to the five considerations in favor of such discrimination mentioned above: (1) that disabled patients have shorter expected lifespans, (2) that disabled patients have lower quality of life, (3) that disabled patients have lower expected economic productivity, (4) that a treatment is less effective for disabled than non-disabled patients, and (5) that a treatment is costlier for disabled than for non-disabled patients.

We expected at least one of the reasons provided to reduce moral condemnation relative to control, but did not have strong a priori hypotheses as to the relative strength of each consideration.^2^

## Materials and Methods

We conducted our experiment in July 2023 as part of an online survey administered by the survey agency YouGov. We recruited a sample of 1,100 respondents from the UK, providing more than 99 percent power to detect a conventionally small effect size (f = .2) in an omnibus test. The UK setting was chosen because, given the prominence and longevity of NICE in the UK health care system, respondents are more likely to be familiar with the use of CEA in health care priority settings. The respondents were quota sampled to be largely representative of the adult population in terms of age (M = 48.6, SD = 17.5, range = 18–91), gender (53% female), geographical region of residence, income, and education. Respondents were remunerated by YouGov in accordance with their usual procedure and rates.

We employed a between-subjects design for our experiment. All the respondents are first presented with a brief text on cost-effectiveness based priority setting.

Because resources are limited, health-care providers must decide how to deploy resources in an efficient way. Sometimes, to this end, they use cost-efficiency analysis. In a cost-efficiency analysis, the costs and benefits, including health benefits, of providing a treatment are given a monetary value, allowing for a comparison of the cost effectiveness of various treatments. When assigning a monetary value to the benefits of a treatment, cost-efficiency analysis considers, for example, how much patients’ quality of life is expected to increase because of the treatment, for how many years of life.

Importantly, this introduction to what cost-effectiveness is and why it is undertaken stresses limited healthcare resources as a prime driver for why this is undertaken.

Respondents were then randomly assigned to one of seven vignettes, all of which describe a case of priority setting. In every vignette, two people need treatment for the eye disease cataracts.^3^ One of the persons also has a mobility disability, arthritis, and this person is not offered treatment for cataracts for reasons of cost-effectiveness. The vignettes are similar regarding the outcome (i.e., who is not offered treatment) and the diseases they describe. They differ, however, in terms of the reasons offered as justification for the decision. Here is the part of the vignette that is identical across all vignettes.

The Birmingham Royal Hospital.

Two patients, Adam and Bob, both approach the Birmingham Royal Hospital in need of cataract eye surgery. Adam and Bob need the surgery to the same degree. If not treated, the cataract will result in slowly worsening of sight and may cause blindness. Now, as it turns out, Bob but not Adam also suffers from the mobility disability, arthritis. Arthritis causes lower life-expectancy, reduced quality of life and lower degree of employability. Thus, other things equal, treatments offered to arthritis patients (when the treatment is unrelated to the arthritis) is less cost-effective than the same treatment offered to non-disabled patients. For this reason, the hospital decides that Adam but not Bob is eligible for cataract eye surgery.

Thus, this case, designed to closely resemble cases discussed in the ethical literature, presents the respondents with a story about how, for reasons of cost-effectiveness only one of them is offered treatment. Respondents in the control conditions saw nothing further, but other respondents were presented with **one** of the following justifications for the decision: “In their assessment, the decision-makers emphasize that” … (the bold text was not highlighted to respondents):

1. As an effect of their disability, patients with arthritis have **shorter expected lifespans** than patients without it.
2. As an effect of their disability, patients with arthritis have **lower quality of life** than patients without it.
3. As an effect of their disability, patients with arthritis will **be less productive of economic benefits** to the good of society than patients without it.
4. The treatment for cataract is **less effective** for patients with arthritis than for patients without it.
5. The treatment for cataract is **more complicated and therefore more expensive** for patients with arthritis than for patients without it.

These variations reflect the reasons highlighted in the ethical literature as some that may make it more permissible to discriminate against people with a disability on the basis of cost-effectiveness analysis. They are thus designed to see if the reasons stressed in that literature would influence assessments of a case similar to many of the cases discussed in the ethical literature. We also included a control vignette where no particular reason is emphasized and, for exploratory reasons, a second control vignette that also did not emphasize any reason but employed the word “condition” instead of “disability”. After the respondents had read the vignette, they were presented with a measure of perceived moral wrongness consisting of six items, which were answered on a 1–7 Likert scale. This scale had acceptable internal reliability (α = .905) and was used as our dependent variable.

## Results

Participants’ judgments of the moral wrongness involved in the hospital’s decision were consistently high across all vignette conditions. This indicates a general disapproval of deprioritizing patients with disabilities based on cost-effectiveness. Ratings were given on a 7-point Likert scale, with the higher values indicating greater perceived moral wrongness. Thus, regardless of justification offered, participants generally thought that it was impermissible to deny Bob treatment due to his disability. In the control condition, where no specific justification for the decision was provided, participants expressed strong disapproval (M = 5.81, SD = 1.09). This control condition serves as a baseline for comparison with the other experimental conditions in which specific justifications for deprioritization were introduced. As described the five experimental conditions each emphazise one rationale for not offering treatment to the person with the disability, pertaining to respectively: reduced life expectancy, lower quality of life, reduced economic productivity, diminished treatment effectiveness, or higher treatment cost. Additionally, a secondary control condition presented the scenario using the term “condition” instead of “disability.” As Table 1 shows, judgments of moral wrongness were high across all experimental conditions, with means ranging from 5.52 to 5.81 on our 7-point scale.

**Table 1:** Perceived moral wrongness means and standard deviations for each condition

Perceived moral wrongness (1–7)	Mean	SD
Control	5.81	1.09
Life Expectancy	5.55	1.26
Life Quality	5.74	1.24
Economic productivity	5.70	1.27
Treatment effectiveness	5.62	1.26
Treatment cost	5.52	1.21
“Condition” control	5.63	1.30

Response options range from 1 to 7

While across all experimental groups judgments of moral wrongness remained similarly high, it is interesting to note how the various condition fared. The lowest is the treatment cost condition: M = 5.52, SD = 1.21). The life expectancy condition is very similar: M = 5.55, SD = 1.26. Slightly higher, we have the Treatment effectiveness condition: M = 5.62, SD = 1.26, and the “Condition” control: M = 5.63, SD = 1.30. and closer to the top of the range we have Quality of life condition: M = 5.74, SD = 1.24, and the Economic productivity condition: M = 5.70, SD = 1.27. The highest is the control condition, where no justification is offered. These differences however, are not statistically significant—as we will now demonstrate.

**Fig. 1:**
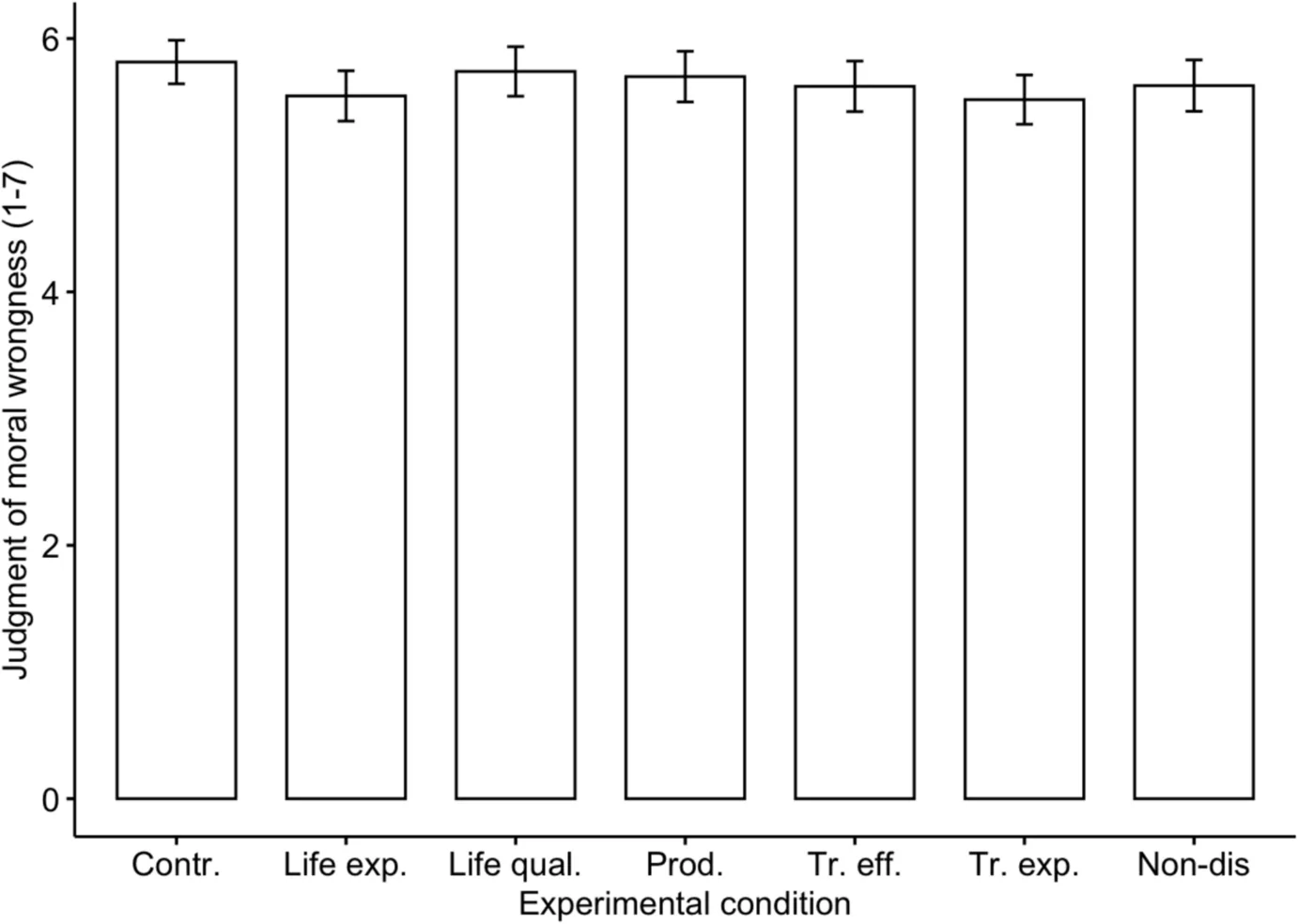
Mean judgment of moral wrongness for each experimental condition. From left to right: control, life expectancy, quality of life, economic productivity, treatment effectiveness, treatment cost, non-discrimination control. Error bars represent 95% confidence intervals

To formally test whether justifications influenced moral judgment, we conducted a one-way ANOVA with perceived moral wrongness as the outcome and experimental condition as the independent factor. There was no significant difference between our experimental conditions on perceptions of moral wrongness (F(6) = 1.141, p = .336). The results are illustrated in Fig. 1. We also ran a planned Tukey post-hoc test directly comparing our control vignette with our “condition” vignette. There was no significant difference between these two conditions (p > .05), suggesting that changing the wording from “disability” to “condition” did not significantly alter participants’ judgments. This comparison will not be discussed further.

The lack of statistically significant differences between the specific justification provided, regardless of whether it appealed to clinical efficacy, economic rationale, or life outcomes, suggests that these different justifications did not substantially affect participants’ views. Rather, participants were uniformly critical of decisions that deprioritized individuals with disabilities in health care allocation, regardless of the rationale.

## Discussion

When comparing the various justifications with the control condition, a clear pattern emerges. The first important insight is that the respondents find this kind of priority setting wrong across the board. The next important finding is that they find the various justifications wrong to a similar extent.

This indicates a general skepticism towards cost-effectiveness-based justifications for disability discrimination. These findings indicate that the general public diverges from the presumptions and discussions prevalent in the ethical literature.

These results are interesting for a number of reasons. They confirm the broad opposition in the general public to cost-effectiveness-based priority setting, which disadvantages people with disabilities [2]. Our study went beyond the existing literature to test whether the nuances pointed out in the ethical discussion of cost-effectiveness and disabilities would affect the assessment. The ethical literature suggests that it would, but our findings indicate the opposite. On the contrary, our findings indicate that the moral reasons provided as justification have little effect on people’s resistance towards discrimination in health care priority-setting.

Of course, there are potential limitations of our study, and here, we wish to outline the most important ones. Given the high mean moral wrongness judgments across all of our experimental conditions, ceiling effects might partially explain the lack of a difference between the various justifications offered in the conditions.

A potential worry is that our basic story seems so unambiguously wrong to respondents that any effect of the justifications is difficult to detect against this backdrop. We note that finding such a strong opposition is interesting in its own right and says something important about the public’s view about disability discrimination and priority setting. However, future work might investigate cases that are morally more ambiguous to laypeople, allowing for greater variability in perceptions across experimental conditions. It is also possible that our choice to mention in the vignette the various reasons why cost-effectiveness may result in lower priority for people with disabilities, may have affected respondents’ views. While this is, of course, the same in each vignette, it is a problem if it affected respondents so much that the variation in which reason is emphasized is not able to influence their assessment.

One reaction to the case used in the vignette is that it provides a too simplistic picture of cost-effectiveness-based priority setting. The concern would then be that these disanalogies might drive resistance to the outcome. Possible disanalogies would be that it is, after all, usually not the hospital that makes the decision and that the decisions made are not between patients but rather treatments. It is so in the sense that priority setting choices based on cost-effectiveness are rarely made between patients, but rather between treatments (and then, therefore, indirectly between groups of patients). In such choices, the costs and benefits may very well be affected by the fact that a certain treatment targets a group of people with disabilities—and indeed in ways similar to those described above.^4^

While we acknowledge the merits of such concerns, there are several reasons for preferring the case at hand. The first is that it is easier for the respondents to judge a case that is about what happens at a hospital as opposed to introducing them to the work of a priority-setting committee. The second reason is that these stylized two-person cases are the exactly the kind of cases employed in the ethical discussion of priority setting. As the paper is primarily written to inform these debates, it makes sense to take this setting as a point of departure. Doing so also means that participants in these ethical debates, who clearly do believe that the various reasons for giving lower priority should matter in exactly these kinds of two-person case, should take note of these findings. In supplement to what has just been said, the distinction between choosing between patients and choosing between treatments is not as clear as it may sound. Many treatments are for very specific (sub)groups of patients. When people in such a group are people with disabilities, the problem of disability and cost-effectiveness resurfaces. Naturally, this defense of our choice of stylized cases does not mean that future work would not benefit from investigating lay judgments of more realistic cases.

There are other possible problems with the design of the vignette. It may have affected people’s responses that the treatment in question (cataract surgery) is a comparatively inexpensive intervention, so that the costs to availability of health care in avoiding disability discrimination might have been perceived as insignificant. It may also have played a role for some respondents that the stated negative effect of the disability in question (arthritis) on life expectancy, as well as on medical cost and effectiveness can be questioned. We acknowledge these as limitations of the study but maintain that our findings contribute to the general understanding of the moral justifiability of disability discrimination in health care priority setting.

Using a UK sample, we found that the respondents were generally against cost-effectiveness-based priority setting when this led to the deprioritization of people with disabilities. This assessment was not significantly changed by our experimental variation emphasizing different reasons for why the person with the disability had received lower priority. One might worry that this conclusion is overly strong, and in part an effect of the presented treatment (cataract surgery) being a non-urgent priority scenario compared to cases involving life-saving treatment and emergency rationing. We agree that these are indeed very different rationing situations and that it is a limitation of our study that it does not inform us about the latter type of cases. However, we find no reason (from ethical theory or speculation) to expect that people would be more positive towards disability discrimination in more emergency-like rationing situations.

The acknowledged limitations of our study point to avenues for future research into this question. Of course, the study could be conducted in countries other than the UK, but we believe it to be even more important that other variations of our approach are tested in future research. One suggestion would be to conduct a similar study that provides a different description of cost-effectiveness analysis (one that does not go through the reasons we do), and another interesting variation would be to conduct a study that seeks to describe the priority-setting decision as one made by a priority-setting institution instead of a hospital, to make the decision even more similar to those actually being made (though, then, less similar to the cases discussed in the ethical literature). A third important variation for future studies would be to conduct experiments, where the seriousness of the disease is different from the cataract eye surgery employed by our study. Conducting the study in a context of life-saving treatment, such as the allocation of ventilators during the COVID-19 pandemic would provide an important variation. As would conducting it with diseases less serious than cataract.

These suggested studies would, of course, come with their own limitations, but completing them would, together with this study, add important pieces to the broader picture of our understanding of the general public’s views on priority setting, cost-effectiveness, and disability.

## Supplementary Information

Below is the link to the electronic supplementary material.

Supplementary file1 (DOCX 16 kb)

## Data Availability

The study involved a survey experiment with voluntaryand anonymous respondents. Respondents were remunerated by YouGovin accordance with their usual procedure and rates. Restrictions apply tothe availability of these data, which were used under licence for the currentstudy and so are not publicly available. The data are, however, availablefrom the authors upon reasonable request.

## Footnotes

1 The available empirical evidence suggests that both harm and disrespect are important [35].

2 Because of the partly exploratory nature of the study, we did not preregister any hypotheses.

3 We verified that there were no significant differences in age, gender, or education level across conditions. This supports the assumption that randomization was successful and that the groups were comparable on key demographic characteristics.

4 However, note that contemporary cost-effectiveness calculations do not take non-health effects into account, such as the likelihood of employment.
