# Supplementary material for: The Moral Justifications of Disability Discrimination in Health Care Allocation: An Experimental Assessment
Source: Health Care Anal. 2025 Aug 22;34(3):666–80. doi: 10.1007/s10728-025-00535-0 (PMC13675370; doi:10.1007/s10728-025-00535-0)
Supplement: Supplementary file 1 [file 10728_2025_535_MOESM1_ESM.docx]

**Supplementary materials for The Moral Justifications of Disability Discrimination in Health Care Allocation: An Experimental Assessment**

1. **Intro text**

[Intro] We are interested in your attitudes about how health-care providers prioritise which treatments to offer to which patients.

Because resources are limited, health-care providers must decide how to deploy resources in an efficient way. Sometimes, to this end, they use cost-efficiency analysis. In a cost-efficiency analysis, the costs and benefits, including health benefits, of providing a treatment are given a monetary value, allowing for a comparison of the cost effectiveness of various treatments.

When assigning a monetary value to the benefits of a treatment, cost-efficiency analysis considers, for example, how much patients’ quality of life is expected to increase because of the treatment, for how many years of life.

2. **Experimental Conditions**

**Control condition**

Please read the following story carefully:

Adam and Bob suffer from cataracts, an eye disease that causes your sight to slowly but steadily worsen and can ultimately result in blindness. To stop the process of deteriorating sight and restore their ability to perform everyday activities such as reading and driving, Adam and Bob need eye surgery.

They approach the hospital about this. Adam and Bob differ only in the respect that Bob also has a severe mobility disability called arthritis. Those who suffer from arthritis have decreased life-expectancy, lower quality of life, and are more likely to be unemployed because of the illness. Arthritis is unrelated to the eye disease.

According to the hospital's policy, only Adam is eligible to receive the eye surgery. This decision is based on a cost-efficiency analysis which shows that providing eye surgery for people with cataracts is not cost-effective when the patient also has arthritis.

**Life Expectancy condition**
Same text as control condition followed by ‘In their assessment, the decision-makers emphasise that, as an effect of their disability, patients with arthritis have shorter expected lifespans than patients without it.’

**Life quality condition**
Same text as control condition followed by ‘In their assessment, the decision-makers emphasise that, as an effect of their disability, patients with arthritis have lower quality of life than patients without it.’

Productivity Condition
Same text as control condition followed by ‘In their assessment, the decision-makers emphasise that, as an effect of their disability, patients with arthritis will be less productive of economic benefits to the good of society than patients without it.’

Effectiveness of treatment condition
Same text as control condition followed by ’In their assessment, the decision-makers emphasise that the treatment for cataract is less effective for patients with arthritis than for patients without it.’

**Expensive treatment condition**

Same text as control condition followed by ‘In their assessment, the decision-makers emphasise that the treatment for cataract is more complicated and therefore more expensive for patients with arthritis than for patients without it.’

***Second control condition (no mention of disability)***

Please read the following story carefully:

Adam and Bob suffer from cataracts, an eye disease that causes your sight to slowly but steadily worsen and can ultimately result in blindness. To stop the process of deteriorating sight and restore their ability to perform everyday activities such as reading and driving, Adam and Bob need eye surgery.

They approach the hospital about this. Adam and Bob differ only in the respect that Bob also has a severe mobility-affecting condition called arthritis. Those who suffer from arthritis have decreased life-expectancy, lower quality of life, and are more likely to be unemployed because of the illness. Arthritis is unrelated to the eye disease.

According to the hospital's policy, only Adam is eligible to receive the eye surgery. This decision is based on a cost-efficiency analysis which shows that providing eye surgery for people with cataracts is not cost-effective when the patient also has arthritis.

3. **Moral wrongness scale (answered on a 1-7 Likert scale)**
1. What is your view on the following statement? The hospital discriminates against Bob.
2. What is your view on the following statement? The hospital's decision is disrespectful toward Bob.
3. What is your view on the following statement? The hospital's decision harms Bob.
4. What is your view on the following statement? The hospital's decision is morally wrong.
5. What is your view on the following statement? The hospital deserves blame for its decision.
6. What is your view on the following statement? The hospital unfairly disadvantages Bob.
